# Immune Thrombocytopenia Induced by Helicobacter pylori Infection: A Case Report and Literature Review

**DOI:** 10.7759/cureus.27809

**Published:** 2022-08-09

**Authors:** Haleema Sadia, Sheeraz Abro, Muneeba Ali, Khalid Uddin, Adesola A Agboola, Shehar Bano, Chinyere L Anigbo, Romil Singh

**Affiliations:** 1 Internal Medicine, Khyber Teaching Hospital, Peshawar, PAK; 2 Internal Medicine, Chandka Medical Hospital, Larkana, PAK; 3 Internal Medicine, Foundation University Medical College, Rawalpindi, PAK; 4 Neurology, Henry Ford Health System, Detroit, USA; 5 Pathology and Laboratory Medicine, Dele Medical Hospital, Lagos, NGA; 6 Internal Medicine, University of Health Sciences, Lahore, PAK; 7 Internal Medicine, University of Nigeria, Enugu, NGA; 8 Critical Care, Allegheny Health Network, Pittsburgh, USA

**Keywords:** helicobacter pylori, h. pylori infection, h. pylori treatment, h. pylori gastritis, immune-mediated thrombocytopenia, immune thrombocytopenia (itp)

## Abstract

Immune thrombocytopenia (ITP) is an autoimmune disease characterized by the production of autoantibodies against the platelet surface antigens. ITP is a diagnosis of exclusion and is further categorized into primary and secondary ITP. The etiology of primary ITP is idiopathic, and secondary ITP is caused by infections and autoimmune disorders. Among infectious etiology of ITP, human immunodeficiency virus, herpes virus, and hepatitis B and C virus are common. *Helicobacter pylori* (*H. pylori*) is a rare cause of ITP, and the relationship between ITP and *H. pylori* is highlighted in the literature. We report a case of ITP in an adult female who presented with hematemesis and petechial rash in the lower limbs. Her initial laboratory results demonstrated thrombocytopenia, and the results of her gastric biopsy and stool antigen were positive for *H. pylori*. She was diagnosed with ITP induced by *H. pylori* because additional causes of ITP were not identified. Her clinical improvement and platelet recovery after initiating *H. pylori* eradication therapy were consistent with *H. pylori*-induced ITP.

## Introduction

Immune thrombocytopenia (ITP) is a type of platelet disorder. ITP is an immune-related syndrome presented with isolated thrombocytopenia, leading to an increased bruising and bleeding tendency. Bleeding usually results from transient or persistent low platelet count [1,2]. Secondary ITP can be inherited or acquired and includes all forms of thrombocytopenia except for idiopathic ITP. Among acquired causes of secondary ITP, various autoimmune disorders and chronic infections are more common. The chronic infections that lead to ITP include human immune deficiency virus (HIV), hepatitis C virus (HCV), and Epstein-Barr virus (EBV) [2]. *Helicobacter pylori* (*H. pylori*) can also trigger ITP; however, the association between ITP and *H. pylori* is not widely reported in the literature [3]. The first association between ITP and *H. pylori* was reported in 1998 when an Italian study highlighted an increase in the platelet cell count in more than 70% of the *H. pylori* diagnosed patients treated with eradication therapy [4]. Herein, we report a case of ITP in a patient diagnosed with *H. pylori *infection.

## Case presentation

A 37-year-old female without significant past medical history was brought to the emergency department after bloody vomiting. She reported three episodes of hematemesis, and her blood was bright red in each episode. She denied any history of fever, trauma, alcohol abuse, abdominal pain, melena, heartburn, hematochezia, or nonsteroidal anti-inflammatory drugs (NSAIDs) use. She also complained of multiple episodes of petechial rash and gingival bleeding in the past, for which she did not seek any medical advice.

On admission, she was hemodynamically stable and oriented to time, place, and person. On examination, she had dried blood on her nares and an evolving petechial rash in her lower limbs. Her abdominal, respiratory, and cardiovascular examinations were unremarkable, with no lymphadenopathy noted in cervical, axial, and groin regions. Her results from initial laboratory tests are shown in Table 1.

**Table 1:** The results of the initial laboratory test. MCH: mean corpuscular hemoglobin; MCHC: mean corpuscular hemoglobin concentration; MCV: mean corpuscular volume; LDH: lactate dehydrogenase; ESR: erythrocyte sedimentation rate.

Parameter	Lab value	Reference range
Hemoglobin	12.9	12-16.5 g/dl
MCH	27	27-32 pg
MCV	80.9	80-100 fL
MCHC	29.4	31-35 g/dl
Red cell count	4.1	4.2-5.4 million cells/µL
White cell count	13,200	4,000-11,000/µL
Platelet count	4,000	150,000-450,000/µL
LDH	297	105-333 IU/L
ESR	12	1-13 mm/hr
Haptoglobin	102	41-163 mg/dL

Peripheral blood smear and protein electrophoresis were unremarkable. Iron studies were within the normal range, including folic acid (7.0 ng/ml), vitamin B12 (840 pg/ml), and copper levels (87 mcg/dl). Her chest X-ray was normal, and the abdomen ultrasound showed no hepatosplenomegaly and ascites. The autoimmune screening was not performed because she had no significant medical history. Owing to her low platelet count, a provisional diagnosis of ITP was made, and she was commenced on oral dexamethasone and pantoprazole infusion for concern of acute upper gastrointestinal (GI) bleed. She developed another episode of hematemesis of bright red blood six hours later. She underwent upper GI endoscopy urgently, which revealed diffuse erosive gastritis. She was continued on pantoprazole infusion and her bleeding resolved without further hematemesis. She was also commenced on 40 mg dexamethasone orally for four days and intravenous immunoglobulin for ITP management.

Her serology was negative for HIV, hepatitis B virus, HCV, and herpes virus. She was tested for *H. pylori* infection through an *H. pylori* stool antigen test, and the result was positive. She was also commenced on eradication therapy, which included amoxicillin 1 g twice daily, metronidazole 500 mg three times daily, pantoprazole 40 mg twice daily, and clarithromycin 500 mg twice daily for 14 days. She was discharged in stable condition with a platelet count of 125,000/uL and was evaluated three days later. Her gastric biopsy results from upper GI endoscopy were positive for *H. pylori *infection*.* She reported improvement in her condition with an increased platelet count of 145,000/uL. An increasing trend in platelet count was observed subsequently. She was evaluated one month after completing eradication therapy, demonstrating almost full recovery with a platelet count of 202,000/uL and no bleeding recurrence. Her repeated stool antigen test for *H. pylori* was negative after one month.

## Discussion

ITP is characterized by an isolated decrease in platelet count (<150,000/uL) in the absence of other etiologies or factors that might be associated with thrombocytopenia [1]. ITP is a diagnosis of exclusion and is further categorized into primary and secondary ITP. Secondary ITP is caused by infections and autoimmune disorders, including systemic lupus erythematous disease, thyroid disorders, antiphospholipid antibody syndrome, HCV, and HIV [2,3,5]. A few case studies have reported the relationship between ITP and *H. pylori*. Gasbarrini et al. reported that eight out of 11 patients diagnosed with ITP responded to eradication therapy [4]. Vanegas et al. reported platelet improvements after eradication therapy in patients with *H. pylori* infection; however, there was a variable response to platelet resolution with no response to complete recovery [6]. It is also concerned that either thrombocytopenia severity or disease duration impacts platelet resolution [7]. We have tabulated the cases of ITP that showed full recovery after *H. pylori* eradication therapy (Table 2) [3,5,8-11].

**Table 2:** Summary of published immune thrombocytopenia cases induced by H. pylori infection. HP: *Helicobacter pylori*; M: male; F: female.

Author	Age/sex	Clinical presentation	Investigation	Platelet count before treatment	Treatment	Clinical outcome
Marques et al. [8]	57/M	Petechial rash, gingivorrhagia	HP stool antigen positive	<10,000/uL	HP eradication therapy	151,000/uL after six months and negative stool antigen
Ramachandran et al. [9]	28/M	Hematemesis	Gastric biopsy positive for HP	<3,000/uL	Dexamethasone plus HP eradication therapy	251,000/uL after six months and negative stool antigen
Goto et al. [10]	53/F	Petechial rash, hematemesis	Gastric biopsy positive for HP	24,000/uL	HP eradication therapy	135,000/uL after eradication therapy
Etou et al. [11]	41/F	Hematochezia	Positive urea breath test	10,000/mL	HP eradication therapy	Platelet count improved after eradication therapy
Hill et al. [3]	54/F	Asymptomatic	HP stool antigen positive	47,000/mL	Prednisone plus HP eradication therapy	145,000/ml after eradication therapy
Tiwari et al. [5]	40/F	Melena, bleeding gums, purpura	Gastric biopsy positive for HP antigen	40,000/mL	Steroids plus triple therapy against HP	Normal platelet count after three months

The pathophysiology of ITP induced by *H. pylori* remains uncertain. The proposed mechanisms included molecular mimicry, down-regulation of the endothelial system, and platelet aggregation, which errand the onset or persistence of ITP [12]. The association between ITP and cytotoxin-associated gene A (CagA) protein produced by *H. pylori* has also been demonstrated. Patients with ITP also have decreased platelet-associated immunoglobulin G (PAIgG) levels, and it was proposed that immune-complex formulations occur due to cross-reactivity between these proteins [13]. Proton pump inhibitor (PPI) use has also been implicated as a rare cause of thrombocytopenia. However, all these inferences were reported in case reports. A clinical retrospective study of 468 patients did not highlight any relationship between PPIs and ITP, and using PPIs in acute GI bleeding is recommended [14]. Regardless of an obvious relationship between ITP and *H. pylori*, platelet count resolution to *H. pylori* eradication treatment has demonstrated divergence between clinical studies. Various factors may impact the results and outcomes, such as genetic and environmental factors, *H. pylori* stains, and prevalence of infection [7].

The definitive treatment of ITP mainly comprises immunomodulatory agents, including steroids (prednisone), immunoglobulin therapy, and rarely, salvage splenectomy or monoclonal antibodies (rituximab) [2]. In cases of secondary ITP, the cause of ITP must be treated. *H. pylori* treatment therapy includes PPIs and a combination of amoxicillin, metronidazole, and clarithromycin. The duration of eradication therapy is seven to 14 days, and treatment efficacy is checked after eight-week post therapy through serum platelet count and stool antigen testing [8-10]. Our patient presented with hematemesis, petechia, and thrombocytopenia. Her recovery after eradication therapy confirmed the diagnosis of ITP induced by *H. pylori* infection.

## Conclusions

Our case highlights the possible relationship between ITP and *H. pylori*. Although *H. pylori* is a rare cause of ITP, it should be included among differentials, and diagnosis and evaluation of underlying etiology in ITP are obligatory for appropriate management. Further clinical studies are warranted to explain the unclear pathophysiology of *H. pylori *in ITP and the paradox of platelet response to anti-*H. pylori* therapy.
